# Two-Dimensional Nanomaterials for Peripheral Nerve Engineering: Recent Advances and Potential Mechanisms

**DOI:** 10.3389/fbioe.2021.746074

**Published:** 2021-11-08

**Authors:** Zhiwen Yan, Cheng Chen, Gonzalo Rosso, Yun Qian, Cunyi Fan

**Affiliations:** ^1^ Department of Orthopedics, Shanghai Jiao Tong University Affiliated Sixth People’s Hospital, Shanghai, China; ^2^ Shanghai Engineering Research Center for Orthopaedic Material Innovation and Tissue Regeneration, Shanghai, China; ^3^ Youth Science and Technology Innovation Studio, Shanghai Jiao Tong University School of Medicine, Shanghai, China; ^4^ Max Planck Institute for the Science of Light, Erlangen, Germany; ^5^ Max-Planck-Zentrum für Physik und Medizin, Erlangen, Germany; ^6^ Institute of Physiology II, University of Münster, Münster, Germany

**Keywords:** two-dimensional nanomaterial, peripheral nerve regeneration, nerve scaffold, nerve tissue engineering, angiogenesis, nanomedicine

## Abstract

Peripheral nerve tissues possess the ability to regenerate within artificial nerve scaffolds, however, despite the advance of biomaterials that support nerve regeneration, the functional nerve recovery remains unsatisfactory. Importantly, the incorporation of two-dimensional nanomaterials has shown to significantly improve the therapeutic effect of conventional nerve scaffolds. In this review, we examine whether two-dimensional nanomaterials facilitate angiogenesis and thereby promote peripheral nerve regeneration. First, we summarize the major events occurring after peripheral nerve injury. Second, we discuss that the application of two-dimensional nanomaterials for peripheral nerve regeneration strategies by facilitating the formation of new vessels. Then, we analyze the mechanism that the newly-formed capillaries directionally and metabolically support neuronal regeneration. Finally, we prospect that the two-dimensional nanomaterials should be a potential solution to long range peripheral nerve defect. To further enhance the therapeutic effects of two-dimensional nanomaterial, strategies which help remedy the energy deficiency after peripheral nerve injury could be a viable solution.

## Two-Dimensional Nanomaterials and Peripheral Nerve Engineering

Two-dimensional (2D) nanomaterials have received great interest by the whole research community due to their exceptional electrochemical properties, based on the special character of atom-scale thickness, which allows for the free transfer of electrons on the material surface. Furthermore, 2D nanomaterials exhibit enhanced and tunable electronic, physical and chemical properties due to their distinctive phase, crystallinity, degree of exfoliation, stability and fundamental limitation of thickness (Rohaizad et al., 2021). In addition, as it is the case for the well-known electroactive nature of peripheral nerve tissue (Yao et al., 2021), 2D nanomaterials possess remarkable electrical properties making them ideal candidates for improving the outcomes of peripheral nerve injuries (PNI) (Qian et al., 2019d). Although there are several exciting studies demonstrating that the 2D nanomaterial-based neural regeneration devices could improve the efficiency of peripheral nerve regeneration (PNR) (Table 1) the exact mechanisms underlying this phenomenon remains elusive.

**TABLE 1:** Therapeutic effects of the 2D nanomaterial functionalized nerve scaffold *in vivo*.

2D Nano-material	Concentration (%)	Nerve defect range (mm)	Time after implantation (weeks)	NCV (ms^−1^)	DCMAP (mV)	Angiogenesis marker	Ref.
Graphene oxide	1	15	18	33.4	25.1	CD31, CD34	Qian et al. (2018b)
Graphene oxide	/	10	8	24.8	9.9	/	Zhang et al. (2020)
Carboxylic graphene oxide	/	10	12	39	3.8	/	Chen et al. (2019)
Reduced graphene oxide	1.14	10	12	25.0	/	/	Wang et al. (2019)
Reduced graphene oxide	0.5	10	12	25	2.5	/	Fang et al. (2020)
Graphene	4	18	18 (months)	42.9	34.5	CD34, VEGF	Qian et al. (2021a)
Graphene	5	10	6	/	5.6	Histology	Lee et al. (2020)
Black phosphorus	0.5	20	16	29.5	22.1	VEGF, CD34	Qian et al. (2019b)
Zinc oxide	10	15	18	45.4	22.6	VEGF, CD34	Qian et al. (2020)
Boron nitride	10	15	18	48.2	30.2	CD34	Qian et al. (2021b)

NVC, Nerve conduction velocity; DCMAP, Distal compound motor action potential; VEGF, Vascular endothelial growth factor; CD, Cluster differentiation; /: Not applicable.

In this review, we summarize the applications of 2D nanomaterials in aiding PNR and focus on the mechanism of new-vessel guided regeneration. First, we summarize the major physiological events occurring after PNI. Second, we describe the application of 2D nanomaterials in peripheral nerve engineering and the corresponding therapeutic effects. Third, we analyze the mechanism by that the newly-formed capillaries provide directionality and metabolic support for neural regeneration. Finally, we discuss the use of 2D nanomaterial-based neural regeneration devices as a potential biomedical strategy to improve long range peripheral nerve defects, and how that could help to remedy the energy deficiency after PNI.

## Peripheral Nerve Damage and Regeneration

**FIGURE 1:**
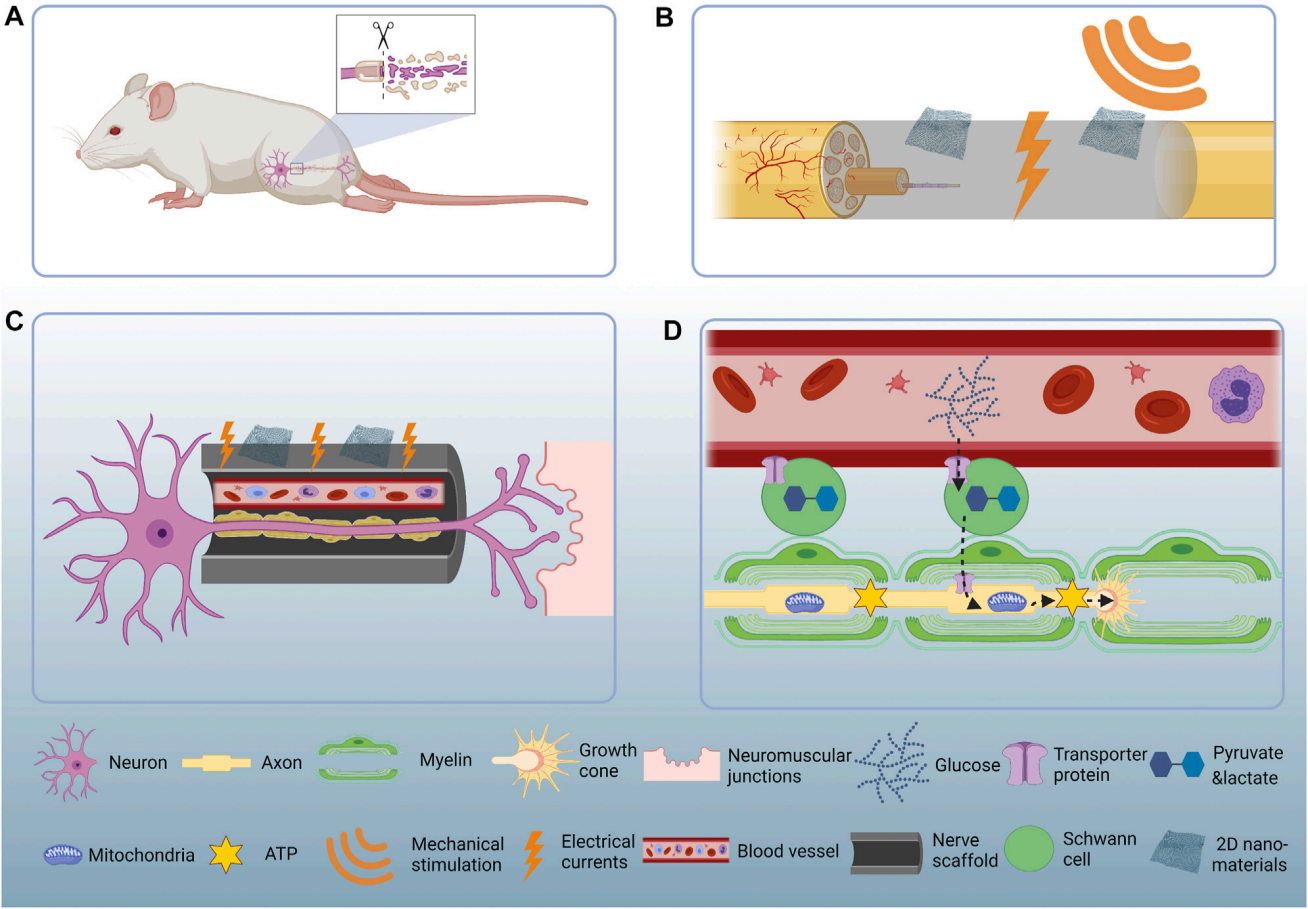
Schematic illustration of the 2D nanomaterial mediated PNR. **(A)** PNI causes sensory and locomotor dysfunction. **(B)** Two-dimensional nanomaterials convert mechanical energy to electrical currents and thereby stimulate PNR. **(C)** Two-dimensional nanomaterials help rebuild the micro-vessels and guide the regenerating nerves back to their original targets. **(D)** Glucose in the reconstructed capillaries get transported to the Schwann cells and then get processed into pyruvate and lactate. The pyruvate and lactate get transported to the regenerating axons and serve as metabolic substrate. The mitochondria accumulate at the axonal growth cone and consume pyruvate and lactate to produce ATP which could be directly used as cellular energy.

The peripheral nerve acts as a real-time information transmitter between human brain and the rest body. Compared to the well-protected brain and spinal cord in the central nervous system by the skull and vertebrates, peripheral nerves are not surrounded by hard structures, making them extremely vulnerable to different physical damage. Normally, PNIs result in numbness, locomotor dysfunction and even life-long disabilities for individuals (Figure 1A), causing huge social and economic burdens (Taylor et al., 2008). The gold standard treatment to PNI (anastomosis and autograft transplanting) have intrinsic limitations such as donor site morbidity and size mismatch (Ray and Mackinnon, 2010). Despite researchers have developed various neural regeneration devices as alternative therapies to PNIs, these products fail to guarantee satisfactory functional nerve recovery.

The peripheral nervous system (PNS) is among the very few tissues in adult mammals which possess remarkable regeneration capabilities after injury. After PNI, Schwann cells–the myelin forming cells in the PNS–dedifferentiate and transform into a repair phenotype that play key roles in the nerve regeneration process (Jessen and Mirsky, 2016; Clements et al., 2017). However, such regeneration process is often mistakenly thought to occur robustly and successfully (Scheib and Höke, 2013). Hence, the complex biological events induced by the injury lead to changes in the biochemical and mechanical properties of the nerve tissue microenvironment, impairing the restoration of nerve function (Yan and Qian, 2020; Qian et al., 2021c). Therefore, it is of tremendous biomedical interest to improve current strategies to regenerate peripheral nerves more efficiently, especially in nerve defect models.

PNIs are characterized by the retraction of two nerve stumps or the direct loss of a nerve segment. During the regeneration process, a natural bridge forms between the two nerve stumps (Min et al., 2021) where damaged axons need to grow through in order to reach the distal nerve trunk and their designated target effectors. The distal trunk, usually referred to as Bands of Büngner, provides a pro-regenerative environment for the outgrowth of axons across the lesion site. However, the environment within the nerve bridge is rather hostile for Schwann cell and axons to travel through. Endothelial cells, which help form new vessels, help remodel the hostile environment and play an essential and inevitable role in the regeneration process (Cattin and Lloyd, 2016). The degree of vascularization determines the success of PNR, i.e., the higher degree of vascularization within the nerve bridge contributes to better PNI outcomes while the inhibition of vascularization destroys normal PNR.

## Applications of 2D Nanomaterials in PNR and the Underlying Mechanisms

In this subsection, we update the applications of 2D nanomaterials for the fabrication of nerve scaffolds, and focus on the mechanism that the 2D nanomaterials could facilitate the formation of new blood-vessels and discuss their pro-regenerative effects for PNR (Figure 1B).

### Graphene

Graphene is the most representative material of the 2D nanomaterial family. The unique nano-scale honeycomb planar structure formed by carbon sp^2^ hybridization, provides graphene with outstanding electrical conductivity, surface area and mechanical properties (Qian et al., 2018c). Convertino et al. observed that dorsal root ganglion (DRG) neurons elongate more when cultured on graphene surfaces compared to glass *in vitro* (Convertino et al., 2020). The authors attribute this effect to the decreased retrograde transport of nerve growth factor (NGF), and suggested that the increased NGF concentration in axons cultured on graphene is responsible for the improved regenerative capacity of peripheral neurons. Furthermore, when encountered with graphene, neurons show hyperpolarized resting membrane potentials detected by patch-clamp technology, indicating that graphene is able to modulate neuron excitability. Furthermore, the decreased neuron membrane charge could be due to the increase of the local hole doping of graphene. An independent study presented by Pampaloni et al. also supports the notion that single-layered graphene could modulate neuron behavior by influencing membrane functions (Pampaloni et al., 2018). Our group fabricated a graphene-based nerve guide to repair a lengthy peripheral nerve gap (Qian et al., 2021a). Results obtained from *in vivo* measurement showed that the vascular endothelial growth factor (VEGF) protein expression within the graphene-based scaffold was higher when compared to the autograft transplantation 18 months after PNI. Interestingly, the robustly formed capillaries provide the regenerating axons with a microenvironment rich in nutrients. The cell soma of peripheral axons resides in spinal cords and DRGs, so we dissected the spinal cords and DRGs of the rats implanted with graphene-based scaffolds 18 months after injury. We found that the expression level of nestin, a protein specifically expressed in neural stem cells that plays essential roles in neural stem cell differentiation, were upregulated. These results indicate that the presence of graphene accelerates axonal regrowth by creating a pro-angiogenesis microenvironment and increase the stemness of neurons. Interestingly, the high VEGF concentration within the graphene scaffold chamber is also responsible for the increased neuronal activity after PNI. The receptors of VEGF are not only expressed on endothelial cells but also present on the axons and growth cones. The conditional knockdown of hypoxia-inducible factor 1a (HIF-1a) in mice DRG leads to impaired neuronal regenerative ability and the local administration of VEGF could partly remedy this (Cho et al., 2015). Sondell et al. also pointed out VEGF stimulates PNR by acting both on the growing axons and cell bodies (Sondell et al., 2000). Therefore, the graphene induced VEGF expression is responsible for both local angiogenesis and neuronal regeneration.

### Graphene Oxide

The incorporation of graphene oxide, an extremely oxidized graphene derivative, elevated the electrical conductivity of a polymeric conduit to 4.55 × 10^4^ S cm^−1^ while the non-oxidized single-layered graphene conduit is 8.92 × 10^−3^ S cm^−1^. We have tested the neural functional recovery from the aspect of electrophysical regain, remyelination degree and morphological regeneration at 6, 12, 18 weeks after implantation (Qian et al., 2018a). Interestingly, the graphene oxide embedded nerve guide shows superior regenerative potential compared to the polymeric counterparts at various time points. Of note, after 18 weeks, the regeneration outcome of graphene oxide containing scaffold was similar to that of the clinical “gold standard” autograft transplantation. Then we proposed that this phenomenon could be due to the pro-angiogenesis effects of graphene oxide. As expected, the micro-vessel density and vessel-like structure area significantly elevated in the graphene oxide containing conduit. Further, the CD34, a transmembrane phosphor-glycoprotein, widely used as biomarker of hematopoietic progenitor cells, significantly increased by the controlled release of graphene oxide (Sidney et al., 2014). Additional results showed that AKT-eNOS-VEGF signaling pathway involved in the increase in endothelial cell proliferation may provide new insights for the underlying physiological mechanisms of graphene function in nerve regeneration.

### Black Phosphorus

Another promising 2D nanomaterials that has recently received tremendous attention is the black phosphorus for its thickness-dependent bandgap, high charge-carrier mobility, in-plane anisotropic structure, and biodegradable properties (Tao et al., 2015). As a new star of the 2D materials family, black phosphorus exhibited huge potential in aiding peripheral nerve regrowth. For example, the incorporation of 0.5% black phosphorus into polymers elevates the electrical conductivity to 9.81 × 10^−3^ S cm^−1^. Hence, a black phosphorus containing nerve scaffold restores the bioelectrical continuity of damaged nerves and promotes the expression of VEGF, thereby contributing to successful formation of new vessels (Qian et al., 2019b). VEGF, as described above, specifically targets endothelial cells and is essential for vasculogenesis. In a rat long-range nerve defect model (20 mm), the incorporation of black phosphorus into a nerve guide has shown to successfully enhance the formation of vessels compared to the polymeric counterparts. The elevated vessel density is responsible for faster neural regeneration. Therefore, the function of target muscle measured by the distal compound motor action potential (DCMAP) increased from 10.1 to 22.1 mV in the polymer group compared to the black phosphorus group 4 months after implantation.

Moreover, mounting evidence suggest that 2D nanomaterials could facilitate PNR by generating electrical currents. Conversely, the process of angiogenesis has also been linked to these 2D materials. Electrical currents that enhance axonal growth combined with the formation of new vessels are two promising outcomes with tremendous potential for the advancement of nerve regeneration strategies. Electrical stimulation has long been proved to be a valid therapeutic strategy for accelerating nerve regrowth (Qian et al., 2019a) and researchers have developed artificial electrical stimulators to facilitate nerve regeneration (Koo et al., 2018). However, such equipment is usually cumbersome to use because it requires external power sources and the implantation of electrodes is also prone to trigger neuroinflammation and gliosis (Cheng et al., 2020). Back in 1991, Fine et al. proposed the application of vinylidenefluoride-trifluoroethylene, a conventional piezoelectrical active material utilized in the engineering of neural regeneration devices (Fine et al., 1991). The piezoelectrical active material possesses the ability to transform the mechanic energy into electrical stimulation. Basically, when the piezoelectrical active neural scaffold gets mechanically deformed by an internal stimulation (e.g., muscle compression) or an external stimulation (e.g., ultrasound), the electrical currents are generated on the scaffold’s surface, thereby providing a wireless and self-powered nerve electrical stimulation therapy. Unfortunately, this innovative experiment encountered some difficulties. The polymeric piezoelectric material was hard to degrade *in vivo* and some fibrous capsules formed around the scaffold (Reis et al., 2012). From a crystallography point of view, except for the cubic class 432, all non-centrosymmetric point groups possess the piezoelectric effect. With the fast development of 2D material synthesis technology, the reduced 2D dimensionality embodies spontaneous breakdown of three-dimensional (3D) symmetry. Therefore, some non-piezoelectric bulk materials may become piezoelectric when thinned to single atomic layer (Lin et al., 2018). In addition, the 2D piezoelectric nanomaterials have the advantage of possessing ultra-high piezoelectric coefficients and the ability to degraded by human enzymes (Fei et al., 2015; Lin et al., 2017).

### Zinc Oxide

Interestingly, it has been shown that the incorporation of 2D zinc oxide embedded in a polymeric conduit is able to bridge a sciatic nerve defect (Qian et al., 2020a). Qian and colleagues took advantage of a treadmill to trigger electrical activity of the zinc oxide nerve implants in rats. Results from these experiments showed that after 30 min running exercise per day during 18 weeks, the zinc oxide group showed significantly improved nerve regeneration outcomes compared to controls animals. Hence, the zinc oxide nerve scaffold promoted the expression of S100 and myelin basic protein (MBP) in regenerated nerve tissues, indicating a higher number of Schwann cells and myelinated axons. In addition, the β3-tubulin and NF160 levels were also elevated, indicating an increased density of regrowing axons. The cellular and molecular mechanisms underlying nerve regeneration in zinc oxide nerve implants remains still poorly understood. However, an *in vitro* study showed that the ultrasonic activated piezoelectric scaffold increased the VEGF secretion in Schwann cells. On the other hand, an *in vivo* study demonstrated that the exercise activated piezoelectric therapy up-regulates the VEGF and CD34 expression levels within the scaffold chamber (Qian et al., 2020).

### Boron Nitride

The incorporation of 2D boron nitride into nerve scaffolds has also shown promising results for the treatment of PNIs. Structured boron nitride possesses unique properties, such as an atomically flat surface, free of dangling bonds, charged impurities, highly chemical stability, superior elastic modulus and outstanding mechanical flexibility (Zhang et al., 2021). Owing to the polarization of the B–N chemical bond, the 2D boron nitride exhibits piezoelectrical activity. Qian et al. incorporated boron nitride into a nerve scaffold to test the neuronal regeneration ability of this 2D nanomaterial (Qian et al., 2021b). The authors first validated that the 2D boron nitride scaffold possessed excellent piezoelectric property measured by piezo-response force microscopy. Then, they applied a treadmill running protocol to induce mechanical deformation on the 2D boron nitride scaffold, thereby generated electrical currents. 18 weeks post implantation, the morphology of regenerated nerve tissues was analyzed by transmission electron microscopy and found a fully regenerated peripheral nerve comprised of axons and myelin sheaths. Specifically, the axons inside the 2D boron nitride scaffold showed an increase of axonal areas, myelin sheath thickness, diameter of myelinated axons, and number of myelinated axons. Furthermore, the locomotor function restoration measured by walking track analysis and DCMAP was improved. The motor function restoration is a challenge for artificial nerve scaffold due to the denervation induced muscle atrophy. The 2D boron nitride scaffold preserved the endplate function and promoted the muscle fiber phenotype shift from slow muscle fiber to fast muscle fiber. The faster axonal regeneration was responsible for this phenomenon and as the micro vessel-reconstruction was the premise for axonal regeneration. As expected, CD34, the indicator of neo-vessel formation, was significantly increased in the 2D boron nitride scaffold group.

## 2D Nanomaterials Facilitate Micro-Vessel Formation and Provide Guidance for Migrating Schwann Cells

It remains controversial whether 2D nanomaterials could enhance the angiogenesis ability of endothelial cells. Cibecchini et al. reported that the 2D graphene oxide compromised the angiogenic potential of primary human endothelial cells *in vitro* when administered at high concentration (50 μg ml^−1^) (Cibecchini et al., 2020). The excessive amount of graphene oxide internalized into the cells forms aggregates and affects the consumption of niacinamide. Excessive graphene oxide hindered angiogenesis of human endothelial cells by altering the distribution of mitochondria and disturbing several metabolic pathways. Contrary to *in vitro* results, *in vivo* data showed that 2D nanomaterials stimulate the formation new vessels in different disease models (Qian et al., 2019d; Norahan et al., 2019; Wierzbicki et al., 2020). One possible explanation for this phenomenon could be that *in vivo* studies utilized scaffolds which allow for the controlled release of 2D nanomaterials. But more importantly, *in vivo* environment possesses the multi-cellular complexity. Macrophages, as the important line of defense in human body, are extremely sensitive to foreign implants such as 2D nanomaterials. Xue et al. pointed out that 2D graphene oxide nanosheets could be phagocytosed by macrophages and activate the toll-like receptors (TLR)/myeloid differentiation factor 88 (MyD88)/nuclear factor kappa-B (NF-κB) pathway, thereby excrete VEGF (Xue et al., 2018). The human umbilical vein endothelial cells (HUVEC) treated with graphene oxide conditioned macrophage culture supernatant exhibited enhanced tube formation ability. Overall, the incorporation of 2D nanomaterials could increase new vessel formation *in vivo*. In the following section we discuss the mechanism behind the micro-vessel mediated PNR.

Back in the 1990s, researchers started to observe the relevance between capillary number and nerve regeneration outcomes. Hobson et al. visualized the interactions between RECA-1 positive endothelial cells, S100 positive Schwann cells and axons at different time points after PNI (Hobson et al., 1997). They found first the sprouted of newly formed blood vessels, followed by the migration of Schwann cells and regenerating axons. Of note, the position of Schwann cells and axons within the nerve bridge never exceed the sprouting vessels. In a different work, the incorporation of the proliferative marker EdU in endothelial cells has been used to prove that the vessels are newly formed (Cattin et al., 2015) and observed that the EdU positive endothelial cells only existed within the regenerating nerves.

How does the endothelial cells respond to the nerve injury? In intact nerve tissues, blood vessels go along with nerve fascicles and supply oxygen and nutrients to maintain the normal physiology of nerve function. However, after traumatic nerve injury, both the nerves and the accompanied blood vessels are severely damaged and eventually completely transected causing a hypoxic microenvironment within the injured site. 2 days after nerve dissection, the injury site undergoes severe hypoxia (Cattin et al., 2015). Interestingly, among the diverse cell components of peripheral nerves, macrophages are extremely sensitive to hypoxia. Over 98% hypooxyprobe-1 positive cells are macrophages and over 80% macrophages are hypoxic. Then, the hypoxic microenvironment stabilizes transcriptional factor HIF-1α and initiates the expression of downstream protein VEGF. The VEGF binds to its receptor on endothelial cells and stimulates endothelial cell proliferation which triggers the formation of new vessels and sprouts from the two ends of dissected nerves to form a vessel bridge.

What is the cellular mechanism behind the blood vessel guided nerve regeneration? Schwann cells, a key cell type that plays a major role in the orchestration of PNR, get attracted by the vessel bridge (Clements et al., 2017). The Schwann cells directly migrate along the vessel bridge in an amoeboid-like mode. The actomyosin cytoskeleton is responsible for this behavior, which is impaired by the application of Rho-kinase inhibitor. Besides, the aligned blood vessels provide directionality for Schwann cell migration and intentionally misdirection (implantation of VEGF releasing beads into the surrounding muscle beds) of endothelial cells results in completely failed PNR. Schwann cells follow the vessel track to form a permissive corridor for regenerating axons to travel through (Cattin et al., 2015). It has been shown that Netrin1/DCC signal acts as a critical cue for regenerating axons to grow alongside the migrating Schwann cells (Webber et al., 2011). Only after the regenerating axons reach their final targets, the peripheral nerve damage could be repaired and the denervation induced atrophy of target organs could be remedied.

Where are the newly formed vessels from? Cattin et al. suggested that the new vessels originate from both the proximal and distal nerve stumps, whereas Hobson et al. pointed out that new vessels originate from the adjacent muscle beds (Hobson et al., 1997; Cattin et al., 2015). The differences in the origin of vessels’ formation could be explained by the different injury models utilized. Cattin et al. used the simple nerve dissection surgery while Hobson et al. implanted a 10 mm fibronectin graft.

In summary, newly formed vessel is the premise for successful PNR. The capillaries directly guide Schwann cell migration and indirectly contribute to axonal regeneration. The ability of angiogenesis modulation should be taken into account when designing a neural regeneration device (Figure 1C).

## The 2D Nanomaterial Facilitated Micro-Vessel Formation Provides Energy for Proliferating Schwann Cells

It remains an open question whether 2D nanomaterials facilitate micro-vessel formation and guidance to migrating Schwann cells. In this subsection, we propose a new mechanism which suggests that the newly-formed capillaries may provide nutrients necessary for the proliferation Schwann cells, and metabolically support neuronal regeneration.

Tissue regeneration is a highly energy-demanding process and energy deficiency happens during PNR as well (Han et al., 2016). In response to injury, the PNS adapts a series of metabolic changes to initiate the regeneration process. Recent studies revealed bioenergetically compensatory processes in neuronal axons and Schwann cells. Mitochondria, the cell power-house in mammalian cells, accumulate at the site of injury in axons by microtubule-based mitochondrial transport from the neuronal soma (Han et al., 2016; Patrón and Zinsmaier, 2016). On the other hand, after nerve injury Schwann cells undergo a glycolysis shift to synthesize excessive amount of pyruvate and lactate (Babetto et al., 2020; Trimarco and Taveggia, 2020). The pyruvate and lactate get transferred to the injured axons, serving as metabolic substrate to support the mitochondrial ATP synthesis (Patrón and Zinsmaier, 2016; Trimarco and Taveggia, 2020). In short, Schwann cells metabolically support axonal regeneration, however, where do Schwann cells get metabolic substrate is not completely understood A similar phenomenon occurs in the central nervous system (CNS) where astrocytes and oligodendrocytes - the glial cells in CNS - transfer lactate to neuronal axons and the lactate is used to generate metabolic energy in the form of ATP, thereby supporting the high energy consumption of neuronal axons (Lee et al., 2012; Rinholm and Bergersen, 2012). Of note, the astrocytes and oligodendrocytes break up blood glucose to generate lactate. In other words, blood vessels energetically support axons through glial cells. As mentioned in *2D Nanomaterials Facilitate Micro-Vessel Formation and Provide Guidance for Migrating Schwann Cells*, the newly formed blood vessel is the premise for neural regeneration in PNS where the capillaries serve as tracks for Schwann cells to migrate along and indirectly guide axonal regeneration (Cattin et al., 2015). It remains open whether the newly formed vessels support axonal regeneration in other ways, for instance, by directly providing energy necessary for axonal regeneration. The latter t is highly possible if we consider that the expression levels of glucose transporter 1 (GLUT1) is upregulated during the glycolysis shift in Schwann cells after PNI (Babetto et al., 2020). The GLUT1 is widely accepted as a protein that help glucose transfer between blood vessels and organs (Veys et al., 2020). Thus, the metabolic substrate Schwann cells use may possibly originated from the nearby blood vessels and angiogenesis supports neurogenesis not only by providing directionality but also by supplying nutrients (Figure 1D).

Therefore, apart from angiogenesis, attention should also be paid upon the metabolic process during PNR. However, several studies pointed out that the 2D nanomaterials pose threat on endothelial cells by disturbing the metabolic pathways. In this regard, Chen et al. performed transcriptional sequencing on 2D black phosphorus exposed aortic artery and identified metabolic disturbance (Chen et al., 2021). Furthermore, Luo et al. also reported that 2D graphene oxide impaired HUVEC viability by compromising lipid droplet biogenesis (Luo et al., 2021). Lipid, as an integral part of cellular membrane, is essential for endothelial cell proliferation. Importantly, the researchers found the addition of oleic acid and α-linolenic acid, metabolic substrate in lipid biosynthesis, could alleviate the 2D graphene oxide induced cytotoxicity in HUVEC and restore the lipid biosynthesis. Overall, 2D nanomaterials facilitate angiogenesis, the newly formed vessels nourish sprouting axons *via* Schwann cells. However, the 2D nanomaterial itself potentially harms the cellular metabolism, while the incorporation of a bio-metabolic active substrate could remedy this and further enhance the therapeutic effects of 2D nanomaterials.

## Final Remarks

Artificial nerve scaffolds hold great promise for the biomedical treatment of injured nerves by connecting the damaged stumps and new nerve tissues form within the scaffold chamber (Liu et al., 2019; Jiang et al., 2020; Yan et al., 2020; Li et al., 2021). However, the functional restoration of PNIs still remains unsatisfactory (Qian et al., 2018b; Qian et al., 2019b; Qian et al., 2019c). The formation of new vessels is the premise for successful PNR, and augmentation of angiogenesis could significantly advance the quality of regenerated nerves and nerve repair strategies. 2D nanomaterials exhibit huge potential in aiding PNR. They not only promote axonal growth *via* electric stimulation, but also facilitate angiogenesis within the regenerating nerve tissue, which directly stimulates Schwann cell proliferation and enhance axonal outgrowth. In this review, we summarize the updates on the mechanism underlying this phenomenon and point out that the 2D nanomaterial promotes PNR *via* facilitating angiogenesis.

Despite there are strong evidences to support that 2D nanomaterials modulate the angiogenesis ability to aid PNR, there are still questions to be answered. What is the target cell of 2D nanomaterials? Whether the 2D nanomaterials directly interact with endothelial cells to drive the proliferation and capillary-forming responses? Or macrophages internalize the 2D nanomaterials to initiate the HIF-1α/VEGF signaling axis, and thereby attract endothelial cell migration? Whether the 2D nanomaterials need to be released from the scaffold to facilitate angiogenesis, or do they take effect *in situ*? Whether the protein within the regeneration microenvironment envelop the 2D nanomaterials to form the “protein corona” and whether the protein-adsorbed nanomaterials change their original biological activities (Wan et al., 2015)?

Moreover, the concentration of 2D nanomaterials should be taken into consideration when fabricating artificial nerve scaffolds. Many nanostructures can facilitate angiogenesis when administered at low dose, but at high dose, they will inhibit new vessel formation (Kargozar et al., 2020). This bimodal effect could be explained by that the low dose nanomaterial triggers moderate level of reactive oxygen species and thereby activates the downstream pro-angiogenesis signaling. However, high dose nanomaterial causes cytotoxicity and compromises cell activity, thereby contributes to the anti-angiogenesis effects.

## Perspective

Although researchers developed various neural regeneration devices to treat PNI, long-range nerve defect remains a huge challenge (Qian et al., 2018a; Zhao et al., 2018; Chen et al., 2020; Qian et al., 2020b; Yan et al., 2021). The possibility for axons to travel through the chamber of a non-functionalized nerve scaffold is under 50% when the nerve defect exceeds 1 cm in rodents (Wieringa et al., 2018). Insufficient vascularization is observed in the long-range nerve defect model and it contributes to poor regeneration outcomes (Farber et al., 2016). With the development of 2D nanomaterials, the compromised vasculature formation could be remedied. Thus, 2D nanomaterials serve as a potential solution to long-range nerve defects.

Apart from angiogenesis, emphasis should also be placed upon the metabolic disturbance after peripheral nerve injury. Recent studies revealed the metabolically regulatory effects of certain biomaterials and the bioenergetic-active materials substantially accelerated tissue regeneration (Liu et al., 2020; Ma et al., 2019). Thus, the incorporation of bioenergetic active substrate into 2D nanomaterial nerve scaffold is poised to be an efficient and effective way to enhance the performance of conventional nerve scaffolds.

PNIs result in over 8.5 million restricted activity days and almost five million bed or disability days per year. In the US alone, over 200,000 peripheral nerve repair procedures are performed annually (Kehoe et al., 2012). So there is a great need for nerve regeneration devices. Since the mid-1980s, the FDA have approved several devices based on natural and synthetic materials to repair nerve defects, however, the therapeutic efficiency remains unsatisfactory. A preclinical assessment was carried out to examine the long-term biosafety and pro-regeneration effects of the graphene loaded nerve conduits (Qian et al., 2021a). The successful translation of these functionalized nerve conduits can meet the huge clinical demand and indirectly relieve the social and economic burden caused by PNIs.
